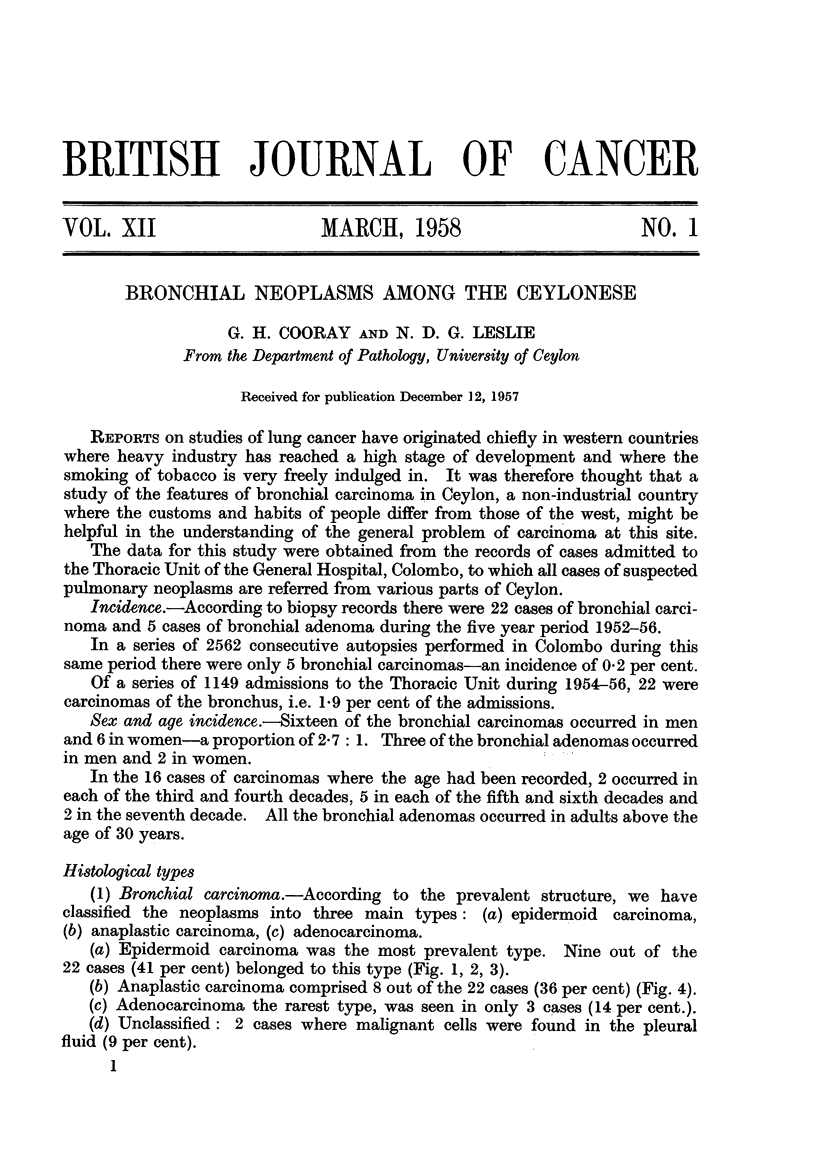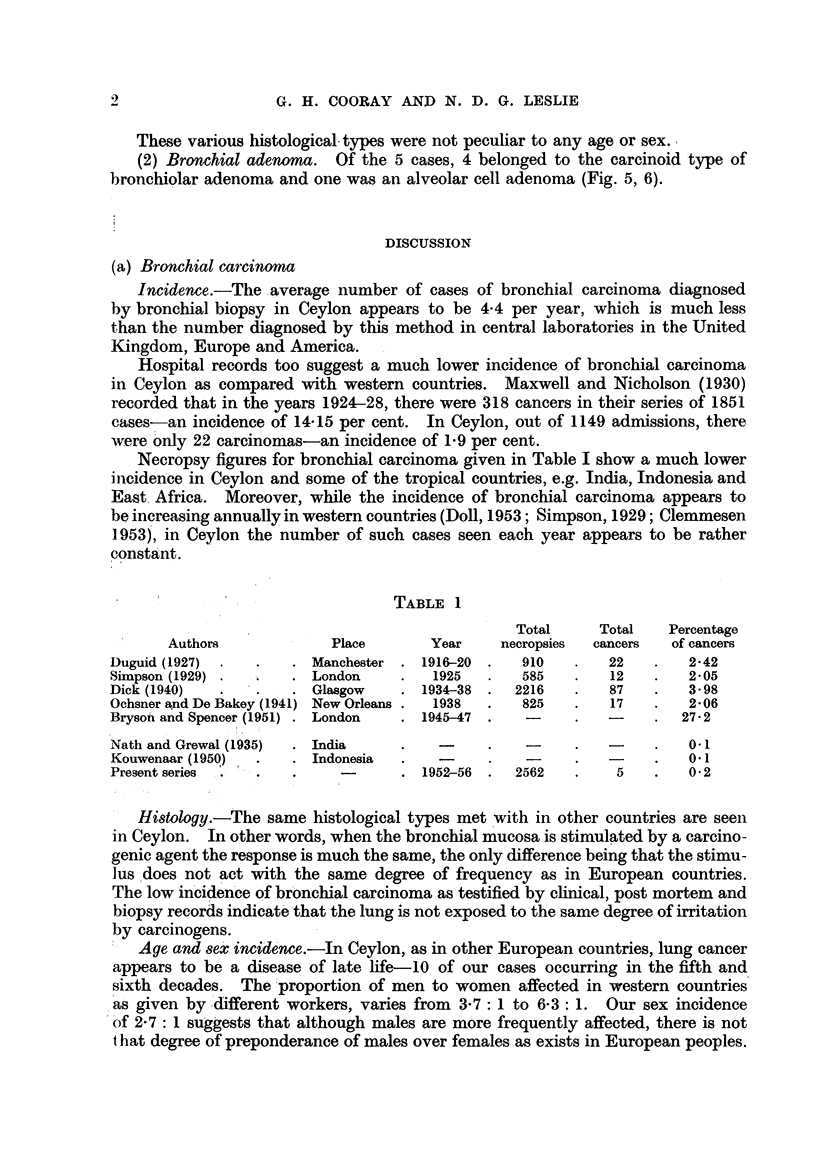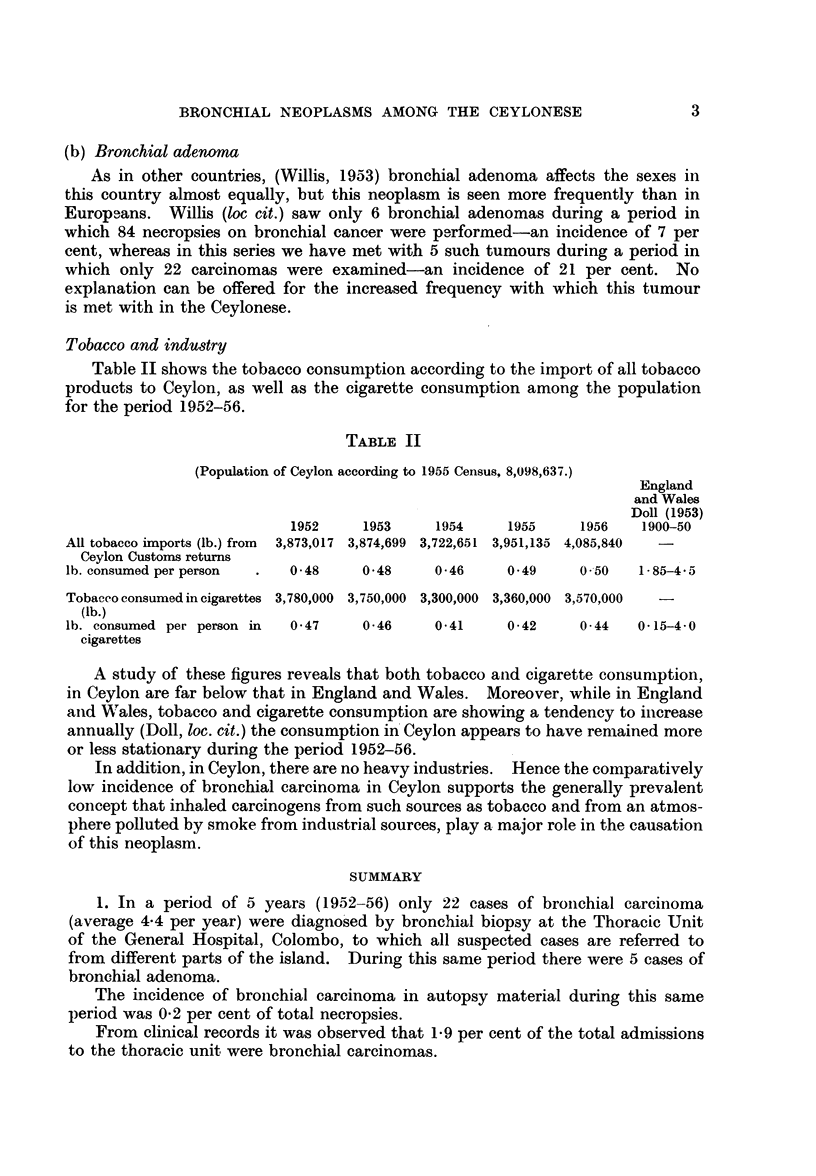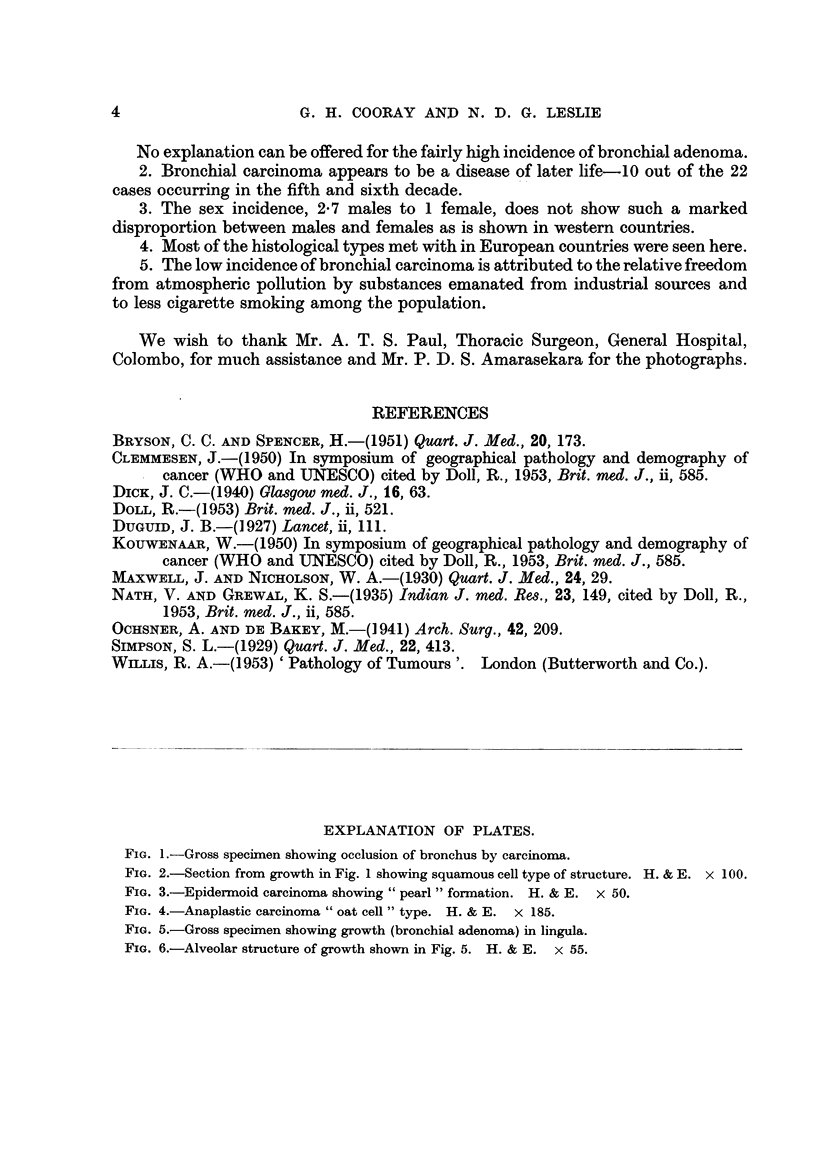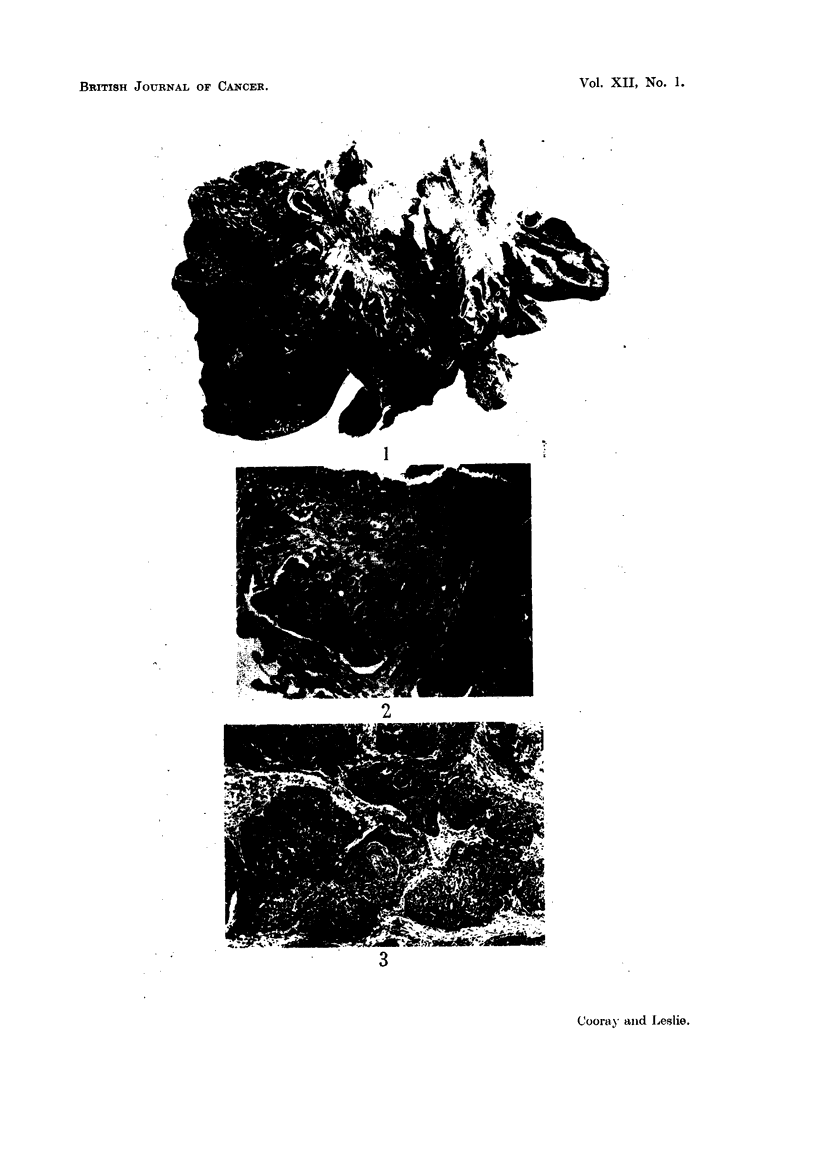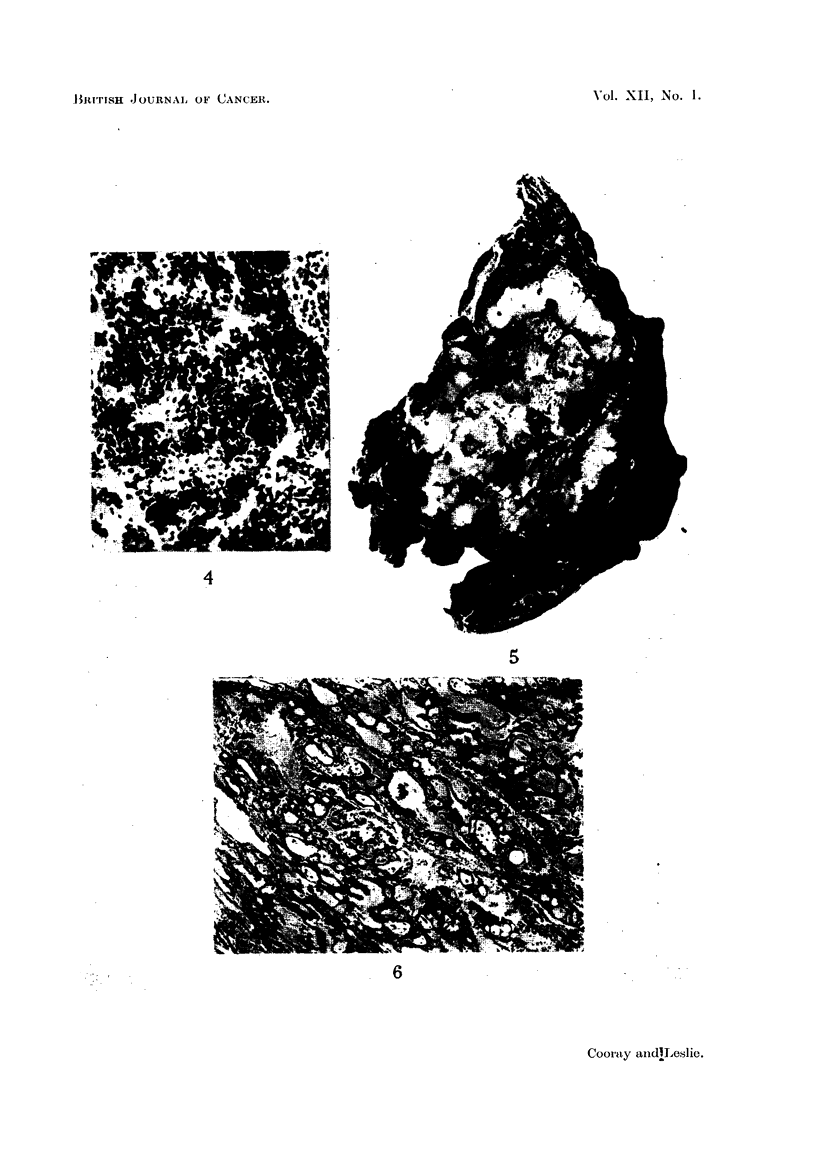# Bronchial Neoplasms among the Ceylonese

**DOI:** 10.1038/bjc.1958.1

**Published:** 1958-03

**Authors:** G. H. Cooray, N. D. G. Leslie

## Abstract

**Images:**


// BRITISH JOURNAL OF CANCER

VOL. XII MARCH, 1958 NO. 1

### BRONCHIAL NEOPLASMS AMONG THE CEYLONESE

G. H. COORAY AND N. D. G. LESLIE

*From the Department of Pathology, University of Ceylon*

Received for publication December 12, 1957

REPORTS on studies of lung cancer have originated chiefly in western countries where heavy industry has reached a high stage of development and where the smoking of tobacco is very freely indulged in. It was therefore thought that a study of the features of bronchial carcinoma in Ceylon, a non-industrial country where the customs and habits of people differ from those of the west, might be helpful in the understanding of the general problem of carcinoma at this site.

The data for this study were obtained from the records of cases admitted to the Thoracic Unit of the General Hospital, Colombo, to which all cases of suspected pulmonary neoplasms are referred from various parts of Ceylon.

*Incidence.*—According to biopsy records there were 22 cases of bronchial carcinoma and 5 cases of bronchial adenoma during the five year period 1952–56.

In a series of 2562 consecutive autopsies performed in Colombo during this same period there were only 5 bronchial carcinomas—an incidence of 0·2 per cent.

Of a series of 1149 admissions to the Thoracic Unit during 1954–56, 22 were carcinomas of the bronchus, i.e. 1·9 per cent of the admissions.

*Sex and age incidence.*—Sixteen of the bronchial carcinomas occurred in men and 6 in women—a proportion of 2·7 : 1. Three of the bronchial adenomas occurred in men and 2 in women.

In the 16 cases of carcinomas where the age had been recorded, 2 occurred in each of the third and fourth decades, 5 in each of the fifth and sixth decades and 2 in the seventh decade. All the bronchial adenomas occurred in adults above the age of 30 years.

### *Histological types*

(1) *Bronchial carcinoma.*—According to the prevalent structure, we have classified the neoplasms into three main types: (*a*) epidermoid carcinoma, (*b*) anaplastic carcinoma, (*c*) adenocarcinoma.

(*a*) Epidermoid carcinoma was the most prevalent type. Nine out of the 22 cases (41 per cent) belonged to this type (Fig. 1, 2, 3).

(*b*) Anaplastic carcinoma comprised 8 out of the 22 cases (36 per cent) (Fig. 4).

(*c*) Adenocarcinoma the rarest type, was seen in only 3 cases (14 per cent.).

(*d*) Unclassified: 2 cases where malignant cells were found in the pleural fluid (9 per cent).

These various histological types were not peculiar to any age or sex.

(2) *Bronchial adenoma.* Of the 5 cases, 4 belonged to the carcinoid type of bronchiolar adenoma and one was an alveolar cell adenoma (Fig. 5, 6).

### DISCUSSION

### (a) *Bronchial carcinoma*

*Incidence.*—The average number of cases of bronchial carcinoma diagnosed by bronchial biopsy in Ceylon appears to be 4·4 per year, which is much less than the number diagnosed by this method in central laboratories in the United Kingdom, Europe and America.

Hospital records too suggest a much lower incidence of bronchial carcinoma in Ceylon as compared with western countries. Maxwell and Nicholson (1930) recorded that in the years 1924–28, there were 318 cancers in their series of 1851 cases—an incidence of 14·15 per cent. In Ceylon, out of 1149 admissions, there were only 22 carcinomas—an incidence of 1·9 per cent.

Necropsy figures for bronchial carcinoma given in Table I show a much lower incidence in Ceylon and some of the tropical countries, e.g. India, Indonesia and East Africa. Moreover, while the incidence of bronchial carcinoma appears to be increasing annually in western countries (Doll, 1953; Simpson, 1929; Clemmesen 1953), in Ceylon the number of such cases seen each year appears to be rather constant.

TABLE 1

| Authors | Place | Year | Total necropsies | Total cancers | Percentage of cancers |
|---|---|---|---|---|---|
| Duguid (1927) | Manchester | 1916–20 | 910 | 22 | 2·42 |
| Simpson (1929) | London | 1925 | 585 | 12 | 2·05 |
| Dick (1940) | Glasgow | 1934–38 | 2216 | 87 | 3·98 |
| Ochsner and De Bakey (1941) | New Orleans | 1938 | 825 | 17 | 2·06 |
| Bryson and Spencer (1951) | London | 1945–47 | — | — | 27·2 |
| Nath and Grewal (1935) | India | — | — | — | 0·1 |
| Kouwenaar (1950) | Indonesia | — | — | — | 0·1 |
| Present series | — | 1952–56 | 2562 | 5 | 0·2 |

*Histology.*—The same histological types met with in other countries are seen in Ceylon. In other words, when the bronchial mucosa is stimulated by a carcinogenic agent the response is much the same, the only difference being that the stimulus does not act with the same degree of frequency as in European countries. The low incidence of bronchial carcinoma as testified by clinical, post mortem and biopsy records indicate that the lung is not exposed to the same degree of irritation by carcinogens.

*Age and sex incidence.*—In Ceylon, as in other European countries, lung cancer appears to be a disease of late life—10 of our cases occurring in the fifth and sixth decades. The proportion of men to women affected in western countries as given by different workers, varies from 3·7 : 1 to 6·3 : 1. Our sex incidence of 2·7 : 1 suggests that although males are more frequently affected, there is not that degree of preponderance of males over females as exists in European peoples.

(b) *Bronchial adenoma*

As in other countries, (Willis, 1953) bronchial adenoma affects the sexes in this country almost equally, but this neoplasm is seen more frequently than in Europeans. Willis (*loc cit.*) saw only 6 bronchial adenomas during a period in which 84 necropsies on bronchial cancer were performed—an incidence of 7 per cent, whereas in this series we have met with 5 such tumours during a period in which only 22 carcinomas were examined—an incidence of 21 per cent. No explanation can be offered for the increased frequency with which this tumour is met with in the Ceylonese.

*Tobacco and industry*

Table II shows the tobacco consumption according to the import of all tobacco products to Ceylon, as well as the cigarette consumption among the population for the period 1952–56.

TABLE II

(Population of Ceylon according to 1955 Census, 8,098,637.)

| | 1952 | 1953 | 1954 | 1955 | 1956 | England and Wales Doll (1953) 1900–50 |
|---|---|---|---|---|---|---|
| All tobacco imports (lb.) from Ceylon Customs returns | 3,873,017 | 3,874,699 | 3,722,651 | 3,951,135 | 4,085,840 | — |
| lb. consumed per person . | 0·48 | 0·48 | 0·46 | 0·49 | 0·50 | 1·85–4·5 |
| Tobacco consumed in cigarettes (lb.) | 3,780,000 | 3,750,000 | 3,300,000 | 3,360,000 | 3,570,000 | — |
| lb. consumed per person in cigarettes | 0·47 | 0·46 | 0·41 | 0·42 | 0·44 | 0·15–4·0 |

A study of these figures reveals that both tobacco and cigarette consumption, in Ceylon are far below that in England and Wales. Moreover, while in England and Wales, tobacco and cigarette consumption are showing a tendency to increase annually (Doll, *loc. cit.*) the consumption in Ceylon appears to have remained more or less stationary during the period 1952–56.

In addition, in Ceylon, there are no heavy industries. Hence the comparatively low incidence of bronchial carcinoma in Ceylon supports the generally prevalent concept that inhaled carcinogens from such sources as tobacco and from an atmosphere polluted by smoke from industrial sources, play a major role in the causation of this neoplasm.

### SUMMARY

1. In a period of 5 years (1952–56) only 22 cases of bronchial carcinoma (average 4·4 per year) were diagnosed by bronchial biopsy at the Thoracic Unit of the General Hospital, Colombo, to which all suspected cases are referred to from different parts of the island. During this same period there were 5 cases of bronchial adenoma.

The incidence of bronchial carcinoma in autopsy material during this same period was 0·2 per cent of total necropsies.

From clinical records it was observed that 1·9 per cent of the total admissions to the thoracic unit were bronchial carcinomas.

No explanation can be offered for the fairly high incidence of bronchial adenoma.

2. Bronchial carcinoma appears to be a disease of later life—10 out of the 22 cases occurring in the fifth and sixth decade.

3. The sex incidence, 2·7 males to 1 female, does not show such a marked disproportion between males and females as is shown in western countries.

4. Most of the histological types met with in European countries were seen here.

5. The low incidence of bronchial carcinoma is attributed to the relative freedom from atmospheric pollution by substances emanated from industrial sources and to less cigarette smoking among the population.

We wish to thank Mr. A. T. S. Paul, Thoracic Surgeon, General Hospital, Colombo, for much assistance and Mr. P. D. S. Amarasekara for the photographs.

### REFERENCES

BRYSON, C. C. AND SPENCER, H.—(1951) *Quart. J. Med.*, **20**, 173.

CLEMMESEN, J.—(1950) In symposium of geographical pathology and demography of cancer (WHO and UNESCO) cited by Doll, R., 1953, *Brit. med. J.*, ii, 585.

DICK, J. C.—(1940) *Glasgow med. J.*, **16**, 63.

DOLL, R.—(1953) *Brit. med. J.*, ii, 521.

DUGUID, J. B.—(1927) *Lancet*, ii, 111.

KOUWENAAR, W.—(1950) In symposium of geographical pathology and demography of cancer (WHO and UNESCO) cited by Doll, R., 1953, *Brit. med. J.*, 585.

MAXWELL, J. AND NICHOLSON, W. A.—(1930) *Quart. J. Med.*, **24**, 29.

NATH, V. AND GREWAL, K. S.—(1935) *Indian J. med. Res.*, **23**, 149, cited by Doll, R., 1953, *Brit. med. J.*, ii, 585.

OCHSNER, A. AND DE BAKEY, M.—(1941) *Arch. Surg.*, **42**, 209.

SIMPSON, S. L.—(1929) *Quart. J. Med.*, **22**, 413.

WILLIS, R. A.—(1953) ' Pathology of Tumours '. London (Butterworth and Co.).

### EXPLANATION OF PLATES.

FIG. 1.—Gross specimen showing occlusion of bronchus by carcinoma.

FIG. 2.—Section from growth in Fig. 1 showing squamous cell type of structure. H. & E. × 100.

FIG. 3.—Epidermoid carcinoma showing " pearl " formation. H. & E. × 50.

FIG. 4.—Anaplastic carcinoma " oat cell " type. H. & E. × 185.

FIG. 5.—Gross specimen showing growth (bronchial adenoma) in lingula.

FIG. 6.—Alveolar structure of growth shown in Fig. 5. H. & E. × 55.